# List of Unregistered Medical Practitioners in Australia and New Zealand

**Published:** 1886-12

**Authors:** 


*List of Unregistered Medical Practitioners in Australia and New Zealand.* Sydney: L. BRUCK. 1886.

Irregular practice seems to thrive more luxuriantly in new countries than with us. We have not now to do with the causes of this, but merely with the facts as brought before us in this small publication. Many in the list profess to be qualified, and allege certain degrees and diplomas; although it is probable that, if investigated, most of these would be found to have no more value than that of the enterprising herbalist, who when asked to explain the right by which he added M.B. after his name, said the letters only stood for

Medical Botanist. A Sydney practitioner advertises that at his lectures "during the evening the Dr. will sing his local song on the Chinese question, and give an unfailing recipe for the growth of the hair. Ladies and gentlemen matrimonially mated on the stage, and other evidences given of the wonderful science of phrenology." Another of Sydney styling himself "Doctor" sells "The Amora Strengthening Wash and Regenerator," which "strengthens the relaxed condition of the generative organs, and will be a source of relief to married men unequal to the duties imposed upon them; in his own case the result exceeded his most sanguine expectations." A "Specialist" in Auckland advertises: "My first advice to all is—that the very instant you notice anything wrong fly to me, for delay is ruin." A travelling "eminent Oculist, Aurist, etc." in Queensland announces that "he carries with him 2 cwt. of the purest unadulterated vegetable medicines." A "Professor" in Melbourne advertises that "his treatment differs entirely from every other in existence, being quite new and original. Diagnosis intuitive and instantaneous."

Although here we are a long way off this—at all events, amongst qualified practitioners—yet it is well to always remember that no man is a great sinner all at once, and that *facilis descensus Averno.* We should be keenly critical of all deviations from professional rectitude, and should sternly check all attempts made to attract public attention. We are sorely in need of a code of minor morals which, if universally recognised, would raise our profession in public estimation. Such a code would forbid obtrusive and multiplied name-plates; conspicuous carriages and trappings; gratuitous advice, a system much abused and quite uncalled for in places which abound in charities, and often adopted merely with a view to impress those who can pay well; the keeping of open surgeries where professional advice is combined with the sale of tooth-brushes; the hire of a couple of rooms, which are dignified by the title of a dispensary, and free attendance given in some particular branch in which the practitioner hopes thereby to be looked upon as a special authority; the degradation of the profession by the provision of advice and medicine for a sum not much more than sufficient to cover the cost of the latter, if properly prescribed, which would then enable us, by a simple exercise in subtraction, to appraise the former at probably its right value.

These are some of the devices by which the ingenuity of man has brought discredit on a profession that should be noble in its simplicity. In the interests of our common brotherhood, we should discourage them to the utmost of our power.

